# A Worm‐Like Illusion: Fibrin Clot Mimicking Parasitic Infection in a Child With Hematuria

**DOI:** 10.1155/criu/9561616

**Published:** 2026-09-03

**Authors:** Ali Sweidat, Yaser El Hout, Abderrahman El Kadhi

**Affiliations:** ^1^ Department of Urology, Hamad Medical Corporation, Doha, Qatar, hamad.qa; ^2^ Department of Pediatric Urology, Sidra Hospital, Doha, Qatar

## Abstract

Hematuria in children often presents a diagnostic puzzle, especially when cystoscopic findings are unexpected—such as the appearance of worm‐like structures. We report the case of a 13‐year‐old male with recurrent hematuria, initially suspected of having a parasitic bladder infection based on endoscopic findings. Ultimately, histopathology identified the structure as a fibrin clot. This case emphasizes the importance of including fibrin clots in the differential diagnosis of pediatric hematuria and highlights the essential role of pathology in guiding management when visual impressions might mislead.

## 1. Introduction

Pediatric hematuria may stem from various etiologies, including urinary tract infections, structural abnormalities, glomerular disorders, and hematologic conditions such as thrombocytopenia or coagulopathies [1, 2]. Occasionally, endoscopic identification of elongated, mobile intravesical structures raises suspicion for parasitic infections, particularly in endemic areas where conditions like schistosomiasis and filariasis are prevalent [3, 4]. However, even in nonendemic settings, such visual findings can lead to premature conclusions.

Fibrin clots, formed from coagulated blood within the urinary tract, may rarely mimic parasitic organisms in shape and movement under cystoscopy. These deceptive appearances can easily be mistaken for helminths, especially when elongated and mobile [5]. Here, we present a pediatric case where a fibrin clot mimicked a worm‐like intravesical structure, reinforcing the diagnostic complexity of such presentations and the necessity of pathological verification.

## 2. Case Presentation

A 13‐year‐old boy with a medical history of hypoplastic left ventricle status post Fontan procedure, epilepsy, autism spectrum disorder, and idiopathic thrombocytopenic purpura under surveillance presented with recurrent episodes of severe gross hematuria occurring intermittently over the preceding 6 months. His regular medications included enalapril and aspirin for his underlying cardiac condition.

On admission, the patient was noted to have worsening hematuria associated with epistaxis. Laboratory investigations revealed persistent thrombocytopenia, with platelet counts ranging between 99 and 101 × 10^3^/*μ*L, and mild anemia with a hemoglobin level of 113 g/L. Total leukocyte count was within normal limits, and differential counts demonstrated normal eosinophil levels. Renal function tests and coagulation profile, including prothrombin time, international normalized ratio, and activated partial thromboplastin time, were within normal ranges.

Urinalysis revealed gross hematuria with red‐colored urine, 3+ blood, and more than 50 red blood cells per high‐power field. Proteinuria was present (3+), with no evidence of infection, as leukocyte esterase and nitrites were negative and white blood cell counts were minimal on microscopy. Urine culture showed no bacterial growth. Urine testing for *Schistosoma haematobium* was performed and returned negative.

Imaging studies, including renal ultrasound and computed tomography of the abdomen and pelvis, demonstrated no structural abnormalities of the kidneys, ureters, or bladder and no evidence of stones or masses.

The patient subsequently underwent diagnostic cystoscopy under general anesthesia. Endoscopic examination revealed an elongated, worm‐like structure within the bladder lumen surrounded by significant debris. The structure was carefully extracted and sent for pathological examination. A concurrent right ureteroscopy was performed and showed no evidence of active bleeding or anatomical abnormalities.

Gross examination of the extracted specimen revealed an elongated fibrinous structure measuring approximately 10 cm in length. Histopathological analysis demonstrated an organized fibrin clot composed of fibrin and erythrocytes, with no evidence of parasitic organisms or malignancy.

Following removal of the clot, the patient′s hematuria and epistaxis gradually resolved over 9 days during admission, and the patient was discharged without further bleeding episodes. His antiplatelet therapy was subsequently adjusted to reduce the risk of recurrent bleeding.

## 3. Discussion

Hematuria in pediatric patients requires a broad differential diagnosis, often including urinary tract infections, structural abnormalities, glomerular disorders, and hematologic conditions such as thrombocytopenia or coagulopathies [1, 2]. Although blood clots are rather common in gross hematuria, the visual presentation of worm‐like formations within the bladder may cause confusion and may lead to unnecessary and expensive workup, especially in nonendemic regions where parasitic infections are less frequently encountered [3, 5].

In the present case, the cystoscopic visualization of an elongated, serpiginous intravesical structure initially raised suspicion for a helminthic infestation such as schistosomiasis or filariasis. Even though these parasitic infections are not common in developed countries, they remain important considerations in patients who have traveled or recently immigrated from endemic regions [3, 4]. For example, *S. haematobium* resides in the bladder′s venous plexus and may cause complications such as calcifications, pseudopolyps, and even bladder cancer in chronic cases [6].

However, this patient did not have any typical risk factors—no travel to endemic regions, no exposure to freshwater, and no signs of eosinophilia on blood tests, which are commonly associated with parasitic infections [3]. He also demonstrated no systemic symptoms such as fever, weight loss, or malaise, making a parasitic etiology less likely.

Moreover, the clot′s morphology was deceptive. Fibrin clots, particularly in patients with coagulation disorders or platelet dysfunction such as idiopathic thrombocytopenic purpura, can form long, thread‐like structures within the bladder. The patient′s combined risk factors, including chronic thrombocytopenia and aspirin therapy following the Fontan procedure, likely contributed to ongoing microscopic bleeding and clot formation [2, 7]. The anatomy of the ureter and bladder likely molded the clot into a long, worm‐like structure. Under cystoscopic examination within a fluid environment, the structure appeared to move, further enhancing the impression of parasitic motility [8].

Fibrin clots are formed through activation of the coagulation cascade and conversion of fibrinogen into fibrin, typically in response to hemorrhage within the urinary tract [9]. In settings of turbulent urinary flow—common in patients with altered cardiovascular physiology such as Fontan circulation—the clots may elongate into unusual configurations. It is therefore important for pediatric urologists to recognize these mimics, as reliance solely on visual impressions may lead to diagnostic confusion.

Several adult cases in the literature have described similar fibrin clot mimics, often in patients receiving anticoagulation therapy or with hematologic malignancies [5]. However, such reports in the pediatric population remain exceedingly rare. One earlier report described bladder clots resembling worms during cystoscopy, emphasizing that without careful histological examination, fibrin clots can easily be mistaken for intravesical parasites [8]. This highlights the essential role of histopathological evaluation in confirming the diagnosis and preventing inappropriate management.

Had empiric antihelminthic therapy such as praziquantel or albendazole been initiated based solely on visual findings, the patient could have experienced adverse drug reactions, particularly given his cardiovascular and hematological comorbidities [10]. Accordingly, this case demonstrates the importance of a structured, stepwise diagnostic approach incorporating clinical history, epidemiological context, laboratory investigations—including eosinophil count—and ultimately pathological confirmation.

This case also reinforces the importance of sending all suspicious or unusual bladder findings for histopathological confirmation, even when they appear benign or visually identifiable. This is particularly important in patients with communication difficulties, neurodevelopmental disorders, or multiple comorbidities, where clinical history may be limited or unclear.

**Figure 1:**
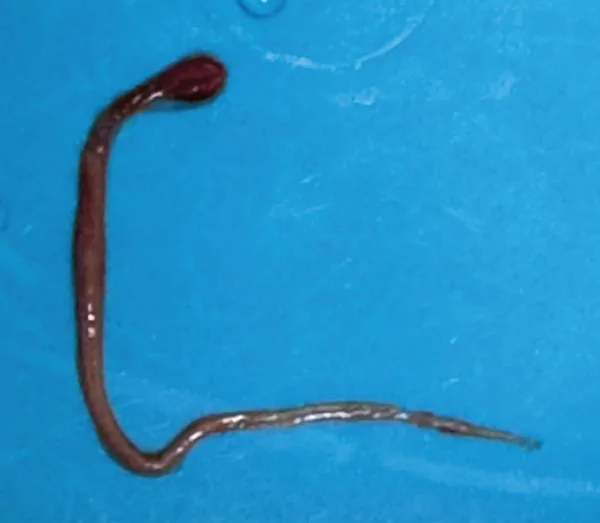
Macroscopic photo of the fibrin clot after removal resembling the parasitic worm.

In addition, this case highlights the important diagnostic role of cystoscopy in pediatric patients with persistent or unexplained gross hematuria despite normal imaging studies. Although ultrasonography and computed tomography are valuable first‐line investigations, cystoscopy allows direct visualization of intravesical abnormalities that may otherwise remain undetected. In selected patients, particularly those with recurrent bleeding or atypical presentations, cystoscopy may help identify clots, inflammatory lesions, foreign bodies, or rare pathologies requiring tissue diagnosis. However, visual findings alone should be interpreted cautiously, as demonstrated in this case where a fibrin clot closely mimicked a parasitic organism (Figure 1) [11, 12].

Finally, we propose that pediatric urology guidelines should emphasize the inclusion of fibrin clots in the differential diagnosis when facing cystoscopic anomalies. Although rare, awareness of this visual mimicry can prevent diagnostic errors and reduce unnecessary interventions.

## 4. Conclusion

This case illustrates how fibrin clots can mimic parasitic organisms in both shape and behavior during cystoscopy, particularly in the pediatric population with complex medical histories. A careful, stepwise approach that includes histopathological confirmation is essential for making an accurate diagnosis and ensuring the best patient care. No matter how convincing visual findings may seem, they should always be backed by microscopic evidence to prevent clinical errors.

## Funding

No funding was received for this manuscript.

## Ethics Statement

Ethical approval was obtained from the Institutional Review Board (IRB) of Sidra Medicine, Doha, Qatar. The IRB granted a waiver of informed consent for publication, provided that no photographs or personal identifiers were included.

## Conflicts of Interest

The authors declare no conflicts of interest.

## Data Availability

All data generated or analyzed during this study are included in this article. Further inquiries can be directed to the corresponding author.
